# ﻿*Grewiakentingensis* (Malvaceae, Grewioideae), a new species from Taiwan

**DOI:** 10.3897/phytokeys.253.141785

**Published:** 2025-03-06

**Authors:** Chou-Yi Lin, Chih-Yi Chang, Chiu-Mei Wang, Hsy-Yu Tzeng, Yen-Hsueh Tseng

**Affiliations:** 1 Department of Forestry, National Chung-Hsing University, No. 145, Hsing-Ta Rd., South Dist., Taichung City, 40227, Taiwan National Chung-Hsing University Taichung City Taiwan; 2 Taiwan Forestry Research Institute, No. 53, Nanhai Rd., Zhongzheng Dist., Taipei City, 10066, Taiwan Taiwan Forestry Research Institute Taipei City Taiwan; 3 Department of Biology, National Museum of Natural Science, No. 1, Guancian Rd., North Dist., Taichung City, 40453, Taiwan Department of Biology, National Museum of Natural Science Taichung City Taiwan

**Keywords:** Critically endangered, *Grewiapiscatorum* Hance, pollen morphology, southern Taiwan, taxonomy

## Abstract

*Grewiakentingensis* Y.H. Tseng, Chih Y.Chang & C.Y.Lin, **sp. nov.**, a new species found on elevated coral reefs in southern Taiwan, is described. The species was previously misidentified as *G.piscatorum* Hance. *Grewiakentingensis* differs from *G.piscatorum* in its habit (procumbent vs. erect to ascending shrub), leaf length (<2 cm vs. up to 7 cm), breeding system (gynodioecious vs. trioecious), smaller flower diameter, fewer stamens, and smaller pollen grains. Color photographs, line drawings, and pollen images of the new species are provided. Additionally, a lectotype for *G.piscatorum* is designated and an identification key for the *Grewia* taxa of Taiwan is presented.

## ﻿Introduction

*Grewia* L. (Malvaceae: Grewioideae) is comprised of 280–300 species, which are distributed in the Old World tropics (Chung et al. 2003, 2005; Randrianasolo et al. 2013). The genus includes trees, shrubs, and climbers, which are characterized by leaves that are 3- to 5-nerved from the base; solitary, cymose, or umbellate inflorescences that are axillary, leaf-opposed, or terminal; and numerous free stamens, an androgynophore, and unlobed or 2- to 4-lobed drupaceous fruits (Liu and Lo 1993; Kubitzki and Bayer 2003; Tang et al. 2007). According to Liu and Lo (1993), four species have been recorded in Taiwan; *G.biloba* G. Don, *G.eriocarpa* Juss., *G.piscatorum* Hance, and *G.rhombifolia* Kaneh. & Sasaki. Chang et al. (2018) later reported an occurrence of *G.tiliifolia* Vahl.

On the basis of floral and fruit morphology, Burret (1926) subdivided *Grewia* into four sections but his classification system is probably artificial (Dorr, pers. comm.). Nonetheless, the Taiwanese taxa can be placed in two of Burret’s sections. *Grewiaeriocarpa* and *G.tiliifolia*, which are characterized by axillary inflorescences, bisexual flowers, entire stigmas lobes, and often unlobed fruits, can be assigned to sect. Axillares Burret while *G.biloba*, *G.piscatorum*, and *G.rhombifolia*, characterized by leaf-opposed, terminal, or rarely axillary inflorescences, unisexual or bisexual flowers, stigmas lobes with filamentary divisions, and usually 4-lobed fruits can be assigned to sect. Glomeratae Burret (see also Chang et al. 2018).

During recent field and herbarium investigations, we noticed that populations of *Grewia* growing on elevated coral reefs in Hengchun Peninsula, Taiwan had been identified as *G.piscatorum* based on sparse hairs on both leaf surfaces and white sepals. However, individuals in this locality diverged from *G.piscatorum* in several characters, including their procumbent habit, smaller stipules, leaves with fewer serrations, smaller flowers, shorter pedicels, and fewer stamens. After careful comparison, we concluded that the material from Hengchun represents a new species, which is described here as *G.kentingensis*.

## ﻿Materials and methods

### ﻿Morphological comparison

We compared our unknown taxon to *Grewiapiscatorum*. Additionally, we compared it to the Taiwanese taxa *G.rhombifolia* and G.bilobavar.biloba as well as to the non-Taiwanese taxa G.bilobavar.parviflora (Bunge) Hand.-Mazz. and G.bilobavar.microphylla (Maxim.) Hand.-Mazz., all of which can be assigned to sect. Glomeratae. Morphological measurements were conducted using both fresh and dried material. Quantitative characters were measured using fresh material, while dry specimens only were used for qualitative character observations and descriptions. For each taxon, at least three individuals were used for measurements and statistical tests. Morphological descriptions follow Liu and Lo (1993); Wahlert et al. (2015); and Barrett (2019).

### ﻿Herbarium resources

Herbarium acronyms from Index Herbariorum were used in this study (Thiers 2024, continuously updated). Voucher specimens collected for the current study were deposited in the herbarium of the Department of Forestry, National Chung Hsing University, Taiwan (TCF). Physical and digital specimens in several herbaria also were examined; physical specimens: Department of Forestry and Natural Resources, National Chia-Yi University, Taiwan (CHIA), Provincial Pingtung Institute (PPI), National Taiwan University, Taiwan (TAI), Endemic Species Research Institute, Taiwan (TAIE), Taiwan Forestry Research Institute (TAIF), TCF, and National Museum of Natural Science, Taiwan (TNM); and digital specimens: SSchool of Life Sciences, Xiamen University (AU), The Natural History Museum (BM), South China Botanical Garden, Chinese Academy of Sciences (IBSC), and the Royal Botanic Gardens, Kew (K).

### ﻿Pollen morphology

Pollen grains were acetolyzed following the method established by Erdtman (1960). Grains were sequentially dehydrated in ethanol and then critical point dried. The dried pollen grains were mounted on a stub and sputter-coated with gold in a Quorum SC7620 sputter coater (Quorum Technologies, Laughton, UK) for 120 s. Subsequently, the grains were observed under a Hitachi S-3400N scanning electron microscope (Hitachi, Ltd, Tokyo, Japan). At least thirty pollen grains were observed and measured for each taxon. The terminology for the morphological descriptions of pollen grains is in accordance with the terminology used by Erdtman (1952) and Halbritter et al. (2018). Information on voucher specimens is provided in Table 1.

**Table 1.** Voucher material for the *Grewia* L. pollen morphology.

Taxon	Location	Coordinate	Altitude	Date	Voucher
*G.kentingensis*	Taiwan. Pingtung County, Hengchun Township, Fongchueisha	21°56'57.8"N, 120°50'16.5"E	73 m	3 May 2024	*C. Y. Lin et al. 69* (TCF)
	Taiwan. Pingtung County, Hengchun Township, Sheding Formosan Sika Deer Restoration Area	21°57'53.0"N, 120°49'39.2"E	154 m	26 June 2024	*C. Y. Lin et al. 93* (TCF)
*G.piscatorum*	Taiwan. Kinmen County, Jincheng Township, Zhaishan Tunnel	24°23'20.0"N, 118°19'07.1"E	20 m	25 May 2024	*C. Y. Lin et al. 80* (TCF)
	Taiwan. Kinmen County, Jinsha Township, Mashan Observation Post	24°31'39.3"N, 118°24'37.9"E	4 m	29 May 2023	*C. Y. Lin 24* (*TCF*)
	Taiwan. Lienchiang County, Nangan Township, Guanmaoshan	26°08'23.7"N, 119°55'44.3"E	15 m	13 June 2023	*C. Y. Lin 26* (TCF)
*G.rhombifolia*	Taiwan. New Taipei City, Wanli Dist., Yehliu Geopark	25°12'32.2"N, 121°41'38.2"E	1 m	22 April 2023	*C. Y. Lin et al. 7* (TCF)
	Taiwan. New Taipei City, Ruifang Dist., Nanyaqiyan	25°07'11.4"N, 121°53'33.1"E	2 m	12 May 2024	*C. Y. Lin 79* (TCF)
	Taiwan. New Taipei City, Ruifang Dist., Bat cave park	25°07'39.2"N, 121°50'05.1"E	4 m	27 June 2024	*C. Y. Lin 96* (TCF)
G.bilobavar.biloba	Taiwan. Hualien County, Xiulin Township, Chongde trail	24°11'34.4"N, 121°39'45.3"E	24 m	26 March 2023	*C. Y. Lin 6* (TCF)
	Taiwan. Kaohsiung City, Mituo Dist., Ta-di Mountain Natural Park	22°46'05.8"N, 120°14'58.7"E	47 m	23 July 2023	*C. Y. Lin 31* (TCF)
	Taiwan. Pingtung County, Chunri Township, Dahan forest road	22°25'13.0"N, 120°40'51.4"E	953 m	11 August 2024	*C. Y. Lin 105* (TCF)

### ﻿Distribution map

A distribution map for this species was generated using QGIS ver. 3.24.2 (QGIS.org 2024) with the package developed by Lin (2018).

### ﻿Data analysis

Quantitative morphological characters of taxa were measured and means and standard deviations were calculated (Table 2). Differences among taxa were analyzed using one-way analysis of variance, followed by Tukey’s Honestly Significant Difference (HSD) multiple-range test (*p* ≤ 0.05) (Oliveira 2013). All statistical analyses were conducted using SPSS ver. 20 (Jasrai 2020).

**Table 2.** Summary of diagnostic characters of Grewiasect.Glomeratae Burret in Taiwan.

Characters		*G.kentingensis*	*G.piscatorum*	*G.rhombifolia*	G.bilobavar.biloba
Habit		procumbent shrub, ca. 5 cm tall	erect to ascending shrub, ca. 1–2 m tall	erect to ascending shrub, ca. 1–2 m tall	small tree, ca. 3–5 m tall
Leaves					
shape		broadly elliptic to elliptic	elliptic, obovate, ovate to rhomboid-ovate	rhomboid, broadly rhomboid to obtrullate	ovate, elliptic to rhomboid-ovate
size (cm)		0.76 ± 0.28^c^ × 0.60 ± 0.25^c^	3.93 ± 1.82^b^ × 2.67 ± 1.31^b^	4.12 ± 1.32^b^ × 3.01 ± 1.23^b^	6.56 ± 2.36^a^ × 3.64 ± 1.26^a^
margin		serrate	serrulate to biserrulate	irregularly serrulate, biserrulate to dentate	serrulate to biserrulate
surfaces		Both surfaces nearly glabrous	Both surfaces nearly glabrous	Both surfaces densely covered with stellate hairs	Both surfaces nearly glabrous
number of serrations		13.83 ± 2.57^c^	47.87 ± 16.00^b^	49.69 ± 17.66^b^	65.48 ± 24.75^a^
stipule length (mm)		0.78 ± 0.19^b^	3.84 ± 0.73^a^	3.99 ± 0.60^a^	4.02 ± 0.79^a^
Sexual system		Gynodioecy	Trioecy	Trioecy	Trioecy
Inflorescences					
position		terminal	leaf-opposed, rarely axillary	leaf-opposed, rarely axillary	leaf-opposed, rarely axillary
per inflorescence flower number		2.39 ± 1.17^c^	9.49 ± 2.63^a^	7.41 ± 2.01^b^	8.42 ± 2.19^ab^
peduncle length (mm)		1.49 ± 0.60^b^	6.63 ± 2.61^a^	6.90 ± 2.64^a^	7.87 ± 2.19^a^
pedicel length (mm)		2.59 ± 1.21^b^	6.05 ± 1.46^a^	6.23 ± 1.35^a^	6.57 ± 1.50^a^
Flowers					
flower diameter (mm)	Bisexual flower	9.49 ± 1.34^c^	16.88 ± 1.71^a^	15.39 ± 1.22^b^	16.25 ± 1.52^ab^
	Female flower	6.75 ± 0.84^c^	11.65 ± 1.29^a^	11.49 ± 1.23^a^	10.09 ± 0.75^b^
sepal number		(3)4–5	(4)5(6)	(4)5(6)	(4)5(6)
sepal size (mm)	Bisexual flower	5.34 ± 0.46^c^ × 1.84 ± 0.27^c^	8.70 ± 0.83^a^ × 3.03 ± 0.44^a^	8.15 ± 0.73^b^ × 2.54 ± 0.43^b^	7.89 ± 0.57^b^ × 2.46 ± 0.27^b^
	Female flower	3.94 ± 0.49^c^ × 1.29 ± 0.23^c^	5.09 ± 0.34^b^ × 1.63 ± 0.13^a^	5.47 ± 0.46^a^ × 1.51 ± 0.13^b^	5.12 ± 0.46^b^ × 1.62 ± 0.12^ab^
petal size (mm)	Bisexual flower	1.32 ± 0.13^d^ × 0.75 ± 0.08^c^	2.65 ± 0.28^a^ × 1.01 ± 0.08^a^	2.32 ± 0.24^b^ × 1.00 ± 0.06^a^	1.79 ± 0.19^c^ × 0.92 ± 0.11^b^
	Female flower	1.37 ± 0.10^c^ × 0.79 ± 0.07^a^	1.93 ± 0.24^a^ × 0.78 ± 0.13^a^	1.64 ± 0.20^b^ × 0.78 ± 0.12^a^	1.87 ± 0.53^a^ × 0.86 ± 0.20^a^
stamens number	Bisexual flower	29.88 ± 3.09^c^	110.86 ± 4.93^a^	82.89 ± 15.88^b^	87.09 ± 18.74^b^
	Female flower	17.55 ± 2.58^c^	79.53 ± 12.30^a^	64.77 ± 8.18^b^	80.67 ± 19.16^a^
filaments length (mm)	Bisexual flower	1.92 ± 0.56^b^	4.02 ± 0.96^a^	3.87 ± 1.16^a^	3.61 ± 0.83^a^
	Female flower	0.76 ± 0.12^c^	1.00 ± 0.27^ab^	0.90 ± 0.28^bc^	1.05 ± 0.37^a^
style length (mm)	Bisexual flower	2.44 ± 0.32^c^	4.18 ± 0.58^a^	3.65 ± 0.22^b^	3.71 ± 0.53^b^
	Female flower	1.83 ± 0.28^c^	2.25 ± 0.22^ab^	2.46 ± 0.21^a^	2.02 ± 0.24^bc^
Druplet size (mm)		4.60 ± 0.22^b^	5.39 ± 0.50^a^	5.43 ± 0.45^a^	5.11 ± 0.36^a^
Pollen					
polar axis (μm)		37.61 ± 1.62^b^	45.32 ± 1.34^a^	45.93 ± 1.65^a^	44.65 ± 1.27^a^
equatorial axis (μm)		28.35 ± 2.47^bc^	29.07 ± 1.05^ab^	29.90 ± 1.38^a^	26.89 ± 1.74^c^
P/E ratio		1.33 ± 0.10^c^	1.56 ± 0.06^b^	1.54 ± 0.11^b^	1.67 ± 0.11^a^
shape		subprolate to prolate	prolate	prolate	prolate
lumen size (μm)		1.98 ± 0.56^b^	2.47 ± 0.77^a^	2.40 ± 0.53^a^	2.24 ± 0.72^ab^
muri width (μm)		0.68 ± 0.06^a^	0.56 ± 0.09^b^	0.55 ± 0.07^b^	0.54 ± 0.06^b^
perforation size (μm)		0.11 ± 0.05^a^	0.11 ± 0.06^a^	0.11 ± 0.06^a^	0.12 ± 0.08^a^
Distribution		Endemic to Taiwan; restricted to elevated coral reefs and coastal grasslands of the eastern coast of Hengchun Peninsula.	Distributed along the coast in Fujian and Hainan provinces in China. In Taiwan, found in Kinmen and Lienchiang counties.	Endemic to Taiwan; distributed along the coast and hills of northern to central Taiwan.	Distributed in central and southern China. At altitudes of ca. 10–1000 m in southern and eastern Taiwan.

^abcd^ Means in the same row followed by the same letter are not significantly different (*p* ≤ 0.05; Tukey’s HSD test).

## ﻿Results and discussion

### ﻿Macromorphological differences

**Figure 1.**
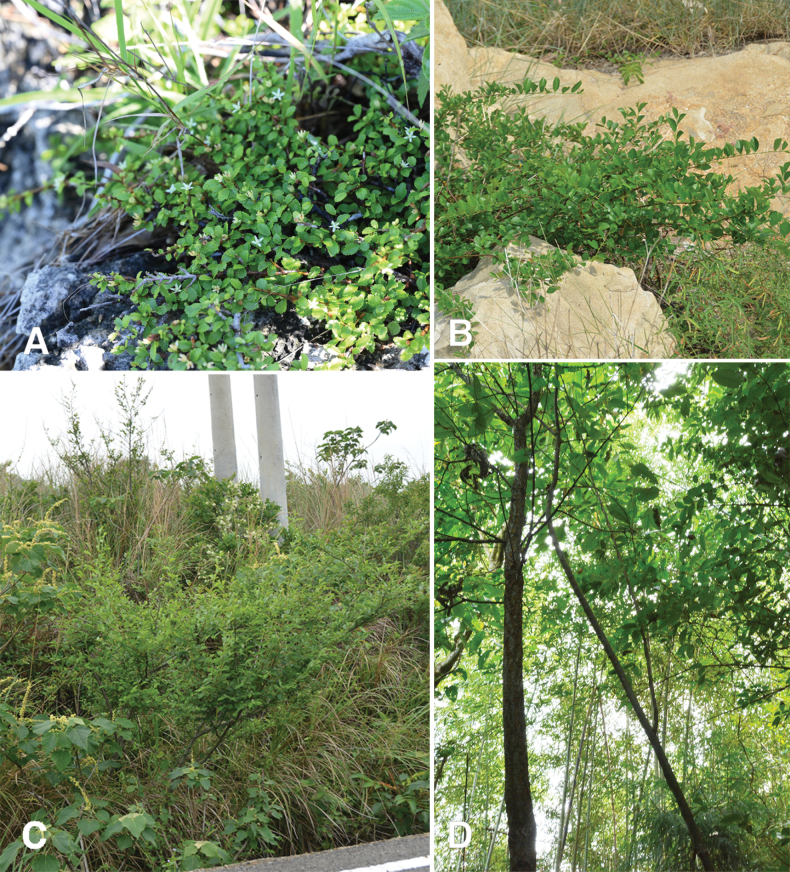
Habit of taxa assigned to Grewiasect.Glomeratae Burret in Taiwan. **A***Grewiakentingensis* Y.H.Tseng, Chih Y.Chang & C.Y.Lin **B***Grewiapiscatorum* Hance **C***Grewiarhombifolia* Kaneh. & Sasaki **D**GrewiabilobaG. Donvar.biloba.

*Grewiakentingensis* can be assigned to sect. Glomeratae in the infrageneric classification system proposed by Burret (1926), as can *G.piscatorum*, *G.rhombifolia*, and G.bilobavar.biloba. The species assigned to sect. Glomeratae in Taiwan have three different habits: 1) G.bilobavar.biloba is a small tree that is ca. 3–5 m tall and usually has a distinct main trunk, 2) *G.piscatorum* and *G.rhombifolia* are erect to ascending shrubs 1–2 m tall, 3) *G.kentingensis* is a procumbent shrub that is clearly distinct from other taxa (Fig. 1, Table 2). Both leaf surfaces in *G.rhombifolia* are densely covered with stellate hairs, whereas both leaf surfaces in *G.kentingensis*, *G.piscatorum*, and G.bilobavar.biloba are nearly glabrous (Table 2). The leaf blades of *Grewia* taxa in Taiwan have distinct shapes: *G.kentingensis* has broadly elliptic to elliptic leaves, *G.piscatorum* displays elliptic, obovate, ovate, and rhomboid-ovate leaves, G.bilobavar.biloba features ovate, elliptic, and rhomboid-ovate leaves, and *G.rhombifolia* has rhomboid, broadly rhomboid, and obtrullate leaves (Table 2). Among the *Grewia* taxa in Taiwan, *G.kentingensis* has the smallest leaves (*p* ≤ 0.05). The leaves of *G.kentingensis* measure 0.4–1.6 × 0.3–1.3 cm. The leaves of *G.piscatorum* measure 0.7–7.8 × 0.5–5.5 cm. The leaves of *G.rhombifolia* measure 1.6–6.7 × 1.1–7.0 cm. The leaves of G.bilobavar.biloba measure 2.1–13.9 × 1.3–7.4 cm (Table 2). *Grewiapiscatorum* exhibits considerable variation in leaf size, partially overlapping with *G.kentingensis* (Table 2). The leaf margin in *G.kentingensis* is serrate, and that of *G.piscatorum* and G.bilobavar.biloba are serrulate to biserrulate. The leaf margin in *G.rhombifolia* is irregularly serrulate or biserrulate to dentate. *Grewiakentingensis* features significantly fewer leaf serrations (9–20) (*p* ≤ 0.05) than *G.piscatorum* (23–82), *G.rhombifolia* (26–88), or G.bilobavar.biloba (29–132) (Table 2).

Significant differences also were observed in the length of peduncles and pedicels among the *Grewia* taxa measured (*p* ≤ 0.05). *Grewiakentingensis* features significantly shorter peduncles and pedicels (peduncles: 0.7–2.7 mm, pedicels: 0.9–4.9 mm) (*p* ≤ 0.05) compared with *G.piscatorum* (peduncles: 3.1–17.2 mm, pedicels: 3.5–8.8 mm), *G.rhombifolia* (peduncles: 3.0–13.1 mm, pedicels: 3.8–8.6 mm), and G.bilobavar.biloba (peduncles: 3.7–14.3 mm, pedicels: 4.4–9.3 mm) (Table 2). Regarding the sexual or breeding system, *G.kentingensis* is gynodioecious, whereas the other taxas are all trioecious (Table 2). Flower diameter in *G.kentingensis* (7.2–11.7 mm in bisexual flowers and 5.2–7.8 mm in female flowers) is significantly smaller (*p* ≤ 0.05) than it is in *G.piscatorum* (14.8–18.5 (–20.2) mm in bisexual flowers and 9.4–14.6 mm in female flowers), *G.rhombifolia* (13.9–17.8 mm in bisexual flowers and 8.9–13.8 mm in female flowers), or G.bilobavar.biloba (13.9–18.7 mm in bisexual flowers and 8.8–11.3 mm in female flowers) (Table 2). Moreover, the sepals and petals in *G.kentingensis* are notably shorter than those in *G.piscatorum*, *G.rhombifolia*, or G.bilobavar.biloba (Table 2). *Grewiakentingensis* also features significantly fewer stamens (25–34 in bisexual flowers and 14–23 in female flowers) (*p* ≤ 0.05) compared with *G.piscatorum* (103–118 in bisexual flowers and 58–102 in female flowers), *G.rhombifolia* (64–103 in bisexual flowers and 51–80 in female flowers), and G.bilobavar.biloba (65–121 in bisexual flowers and 55–98 in female flowers (Table 2). Additionally, the filaments and styles of bisexual flowers in *G.kentingensis* are significantly shorter (*p* ≤ 0.05) than those in other taxa (Table 2).

Finally, although all the taxa assigned to sect. Glomeratae in Taiwan exhibit 4-lobed fruits with nearly glabrous surfaces that turn red after maturation, drupelet size in *Grewiakentingensis* (4.1–5.2 mm) is significantly smaller (*p* ≤ 0.05) than those in *G.piscatorum* (4.4–6.4 mm), *G.rhombifolia* (4.7–6.3 mm), or G.bilobavar.biloba (4.5–5.6 mm) (Table 2).

Other taxa that are morphologically similar to *Grewiakentingensis* and assigned to sect. Glomeratae but not found in Taiwan include G.bilobavar.parviflora, which is distributed from northern to southern China and the Korean Peninsula (Chang 1989; Tang et al. 2007; Chang et al. 2014) and G.bilobavar.microphylla, which is distributed in Sichuan and Yunnan provinces in China (Chang 1989; Wu et al. 1995; Tang et al. 2007). They all exhibit common characteristics such as white sepals, unisexual or bisexual flowers, and 4-lobed drupes that turn red upon maturation (Chang 1989; Tang et al. 2007). However, these two varieties are shrubs or trees 1–4-m tall (Chang 1989; Tang et al. 2007), whereas *G.kentingensis* is a procumbent shrub (Table 3). In addition, *G.kentingensis* can be readily distinguished from G.bilobavar.parviflora by its broadly elliptic to elliptic (vs. ovate) leaf blade shape, smaller leaf blade size (0.4–1.6 × 0.3–1.3 cm vs. 3.0–11.5 × 2.0–7.0 cm), nearly glabrous (vs. abaxial surface covered with stellate hairs) leaf surfaces, and solitary to cymose (vs. umbellate) inflorescences (Bunge 1835; Chang 1989; Tang et al. 2007) (Table 3). The leaf blade size in G.bilobavar.microphylla is smaller (1.0–2.5 × 0.9–1.5 cm), and its range overlaps with that of *G.kentingensis* (0.4–1.6 × 0.3–1.3 cm) (Table 3). Grewiabilobavar.microphylla has ovate leaf blades with an acute to obtuse apex, stellate hairs covering the abaxial surface of leaves, and tomentum on the adaxial surface of its petals (Maximowicz 1889; Chang 1989; Tang et al. 2007). *Grewiakentingensis* features broadly elliptic to elliptic leaf blades with a rounded to obtuse apex, nearly glabrous leaf surfaces, and glabrous adaxial surface on its petals (Table 3).

**Table 3.** Summary of diagnostic characters of *Grewiakentingensis* Y.H.Tseng, Chih Y.Chang & C.Y.Lin and two non-Taiwanese species of sect. Glomeratae Burret.

Characters	*G.kentingensis*	*G.bilobavar.microphylla ^a^*	*G.bilobavar.parviflora ^b^*
Habit	procumbent shrub, ca. 5 cm tall	shrub or tree, ca. 1–4 m tall	shrub or tree, ca. 1–4 m tall
Leaves			
shape	broadly elliptic to elliptic	ovate	ovate
size (cm)	0.4–1.6 × 0.3–1.3	1.0–2.5 × 0.9–1.5	3.0–11.5 × 2.0–7.0
margin	serrate	biserrulate	biserrulate
surfaces	Both surfaces nearly glabrous	adaxial surface nearly glabrous, abaxial surface covered with stellate hairs	adaxial surface nearly glabrous, abaxial surface covered with stellate hairs
Inflorescences	solitary to cyme	umbel	umbel
Adaxial surface of petals	glabrous	tomentose	glabrous
Distribution	Endemic to Taiwan; restricted to elevated coral reefs and coastal grasslands of the eastern coast of Hengchun Peninsula.	Distributed in Sichuan and Yunnan provinces in China.	Distributed in northern to southern China and the Korean Peninsula.

^a^ Character states from Maximowicz (1889), Chang (1989) and Tang et al. (2007). ^b^ Character states from Bunge (1835), Chang (1989) and Tang et al. (2007).

### ﻿Pollen morphology

Pollen grains of taxa assigned to sect. Glomeratae in Taiwan are medium-sized and tricolporate, subprolate, or prolate (Fig. 3, Table 2). These observations are consistent with those provided by Huang (1972) and Hsieh and Huang (1983). *Grewiakentingensis* has a significantly shorter polar axis (*p* ≤ 0.05) (35.8–41.4 μm) than *G.piscatorum* (42.8–47.8 μm), *G.rhombifolia* (42.8–48.2 μm), or G.bilobavar.biloba (42.5–46.9 μm) (Fig. 3, Table 2). The P/E ratio in *G.kentingensis* is the smallest (1.1–1.5) and is significantly lower (*p* ≤ 0.05) than that in the other species (Fig. 3, Table 2). In addition, pollen grains of *G.kentingensis* are subprolate to prolate in shape, whereas those of the other taxa are prolate (Fig. 3, Table 2). The exine in all these taxa is reticulate, with perforations in the lumen (Fig. 3). Perforation size is not significantly different among the four species (Fig. 3, Table 2). Lumen size in *G.kentingensis* (1.0–2.9 μm) is significantly smaller (*p* ≤ 0.05) than that in *G.piscatorum* (1.1–4.0 μm) or *G.rhombifolia* (1.4–3.5 μm). However, no significant difference was observed in the lumen size between *G.kentingensis* and G.bilobavar.biloba (1.1–3.6 (–4.0) μm) (Fig. 3, Table 2). Muri width in *G.kentingensis* (0.6–0.8 μm) is significantly larger (*p* ≤ 0.05) than that in *G.piscatorum* (0.4–0.7 μm), *G.rhombifolia* (0.4–0.7 μm), or G.bilobavar.biloba (0.4–0.7 μm) (Fig. 3, Table 2). In conclusion, *G.kentingensis* pollen can be distinguished from that of other taxa by its shape, smaller polar axis, smaller P/E ratio, smaller lumen size, and wider muri.

**Figure 2.**
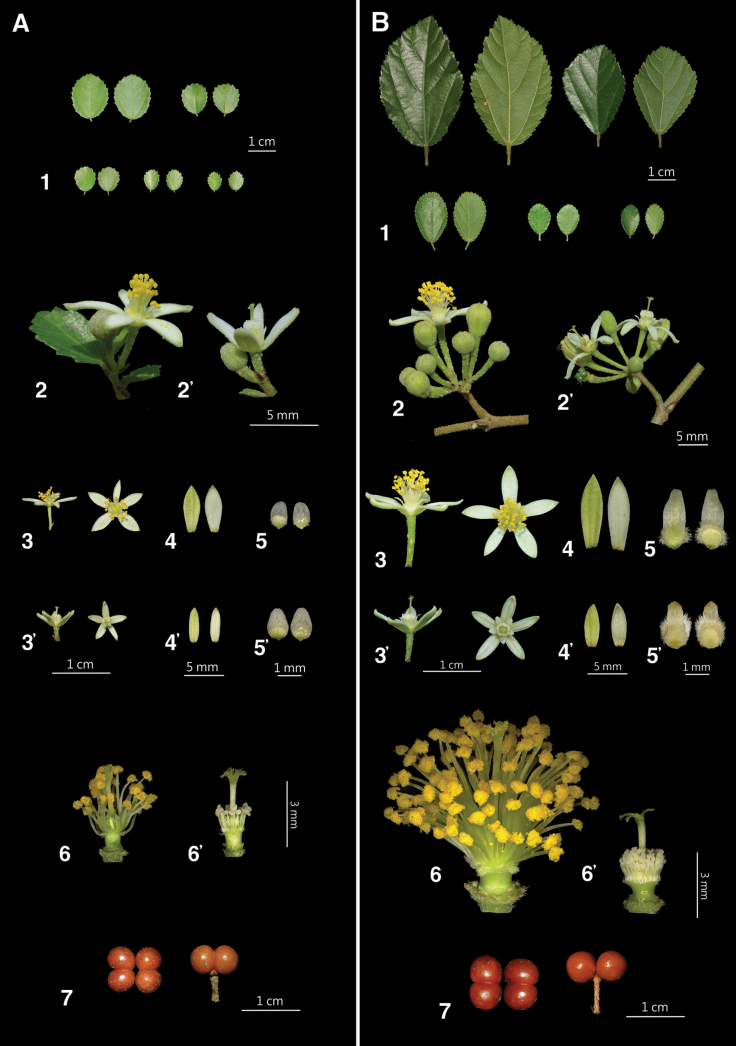
Comparison of morphological characters. **A***Grewiakentingensis* Y.H.Tseng, Chih Y.Chang & C.Y.Lin **B***Grewiapiscatorum* Hance **1** leaves **2** bisexual inflorescence **2**' female inflorescence **3** bisexual flower **3**' female flower **4** sepal (bisexual) **4**' sepal (female) **5** petal (bisexual) **5**' petal (female) **6** bisexual flower (sepals and petals removed) **6**' female flower (sepals and petals removed) **7** fruit (left: overhead view; right: side view).

**Figure 3.**
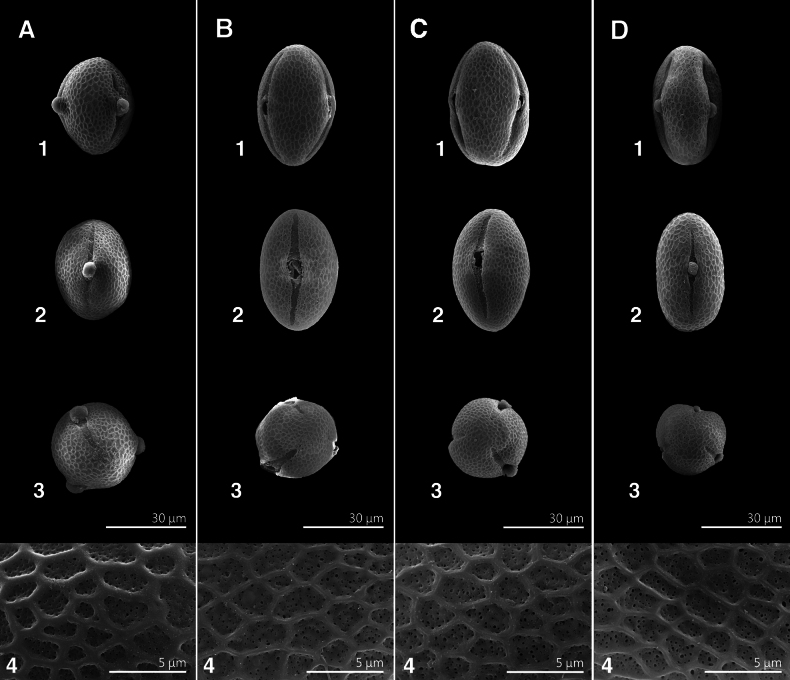
Comparison of pollen morphology of four *Grewia* taxa. **A***Grewiakentingensis* Y.H.Tseng, Chih Y.Chang & C.Y.Lin **B***Grewiapiscatorum* Hance **C***Grewiarhombifolia* Kaneh. & Sasaki **D**GrewiabilobaG. Donvar.biloba**1** equatorial view **2** colporate view **3** polar view **4** surface sculpture.

### ﻿Re-evaluation of *Grewiapiscatorum* in Kinmen and Lienchiang counties, Taiwan

*Grewiapiscatorum* was described by Hance (1866), who based the name on a collection by Swinhoe made on Lamyet Island (Nanjeih Island) in Fujian Province, China (*Swinhoe* in Herb. Hance *6527*). During our field trip to Kinmen and Lienchiang counties in Taiwan and based on examination of specimens, we found that the *Grewia* in Kinmen and Lienchiang counties closely resembled the type specimen of *G.piscatorum*. Additionally, the *Grewia* populations found in Kinmen and Lienchiang counties are geographically close to the type locality of *G.piscatorum*; both counties located on islands along the coast of Fujian Province.

**Figure 4.**
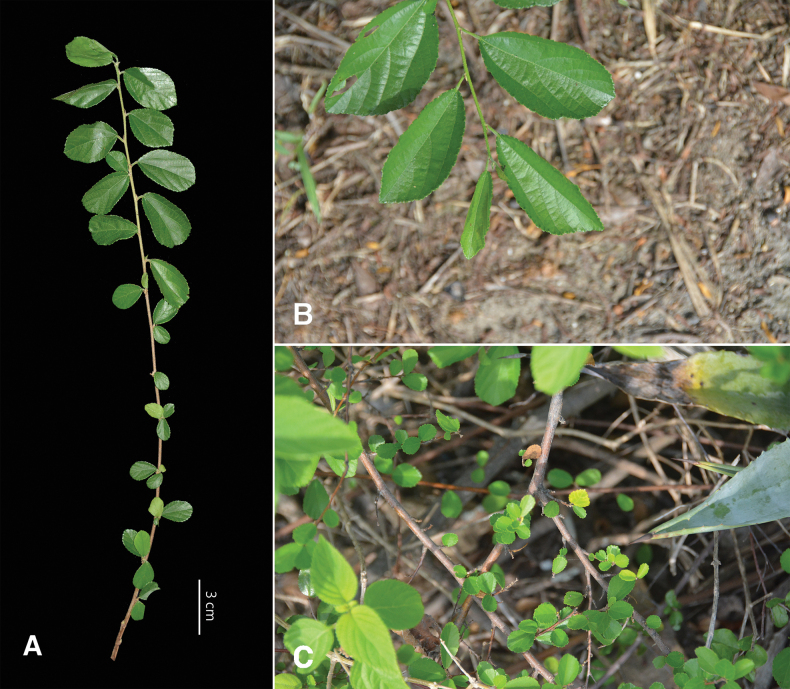
Variation in leaf size of *Grewiapiscatorum* Hance. **A** variation in leaf size at different positions on the same branchlet **B** leaves near the distal end of a branchlet **C** leaves near the proximal end of a branchlet.

In previous studies, the *Grewia* in Kinmen and Lienchiang counties was identified as *G.rhombifolia* (Kuo 2004; Lu 2011). However, we found that this *Grewia* has nearly glabrous leaf surfaces on both sides, with sparse stellate hairs on the veins. Thus, it differs from *G.rhombifolia*, which is characterized by a dense covering of stellate hairs on both leaf surfaces. Hance (1866) stated that *G.piscatorum* has a small leaf blade size. After measuring the type specimen, we determined the leaf blade size to be 0.7–2.5 cm. Nevertheless, based on field observations, we found that leaf blade size varies greatly within an individual plant, differing by leaf position on the branchlet (Fig. 4). On the same branchlet, leaves near the distal end of a branchlet are usually larger, reaching 3–7 cm, whereas leaves near the proximal end of a branchlet are smaller, with a size of 0.5–2 cm. This latter leaf blade size matches that of the type specimen of *G.piscatorum* (Fig. 4). This variation in leaf size has also been found in Bombacoideae (Malvaceae) (Carvalho-Sobrinho et al. 2024). Additionally, such variation in leaf morphology has been observed in *G.rhombifolia*. Smaller leaf blade forms of *G.rhombifolia* in Taiwan have been identified as *G.piscatorum* (Sasaki 1928; Kanehira 1936; Li 1963; Liu and Lo 1993). In previous studies, leaf blade size was often used for identification (Li 1963; Liu et al. 1988; Liu and Lo 1993; Liu et al. 1998). However, variation in leaf blade size can contribute to overlap in size measurements among different taxa thereby rendering the identification of closely related taxa by this criterion alone challenging. In addition to leaf blade size, other characters such as habit, leaf vestiture, and floral morphology should be considered in comparisons for identification (Table 2).

The leaf blade size in *Grewiakentingensis* is much more consistent, with a leaf blade size of 0.5–1.5 cm. Leaves do not exceed 2 cm, which is significantly different from leaves of *G.piscatorum*. In summary, based on the similarity to the type specimen and description of *G.piscatorum* by Hance (1866), we consider the *Grewia* previously identified as *G.rhombifolia* in Kinmen and Lienchiang counties to be *G.piscatorum*.

### ﻿Comparison of the distributions of *Grewiakentingensis* and *G.piscatorum*

**Figure 5.**
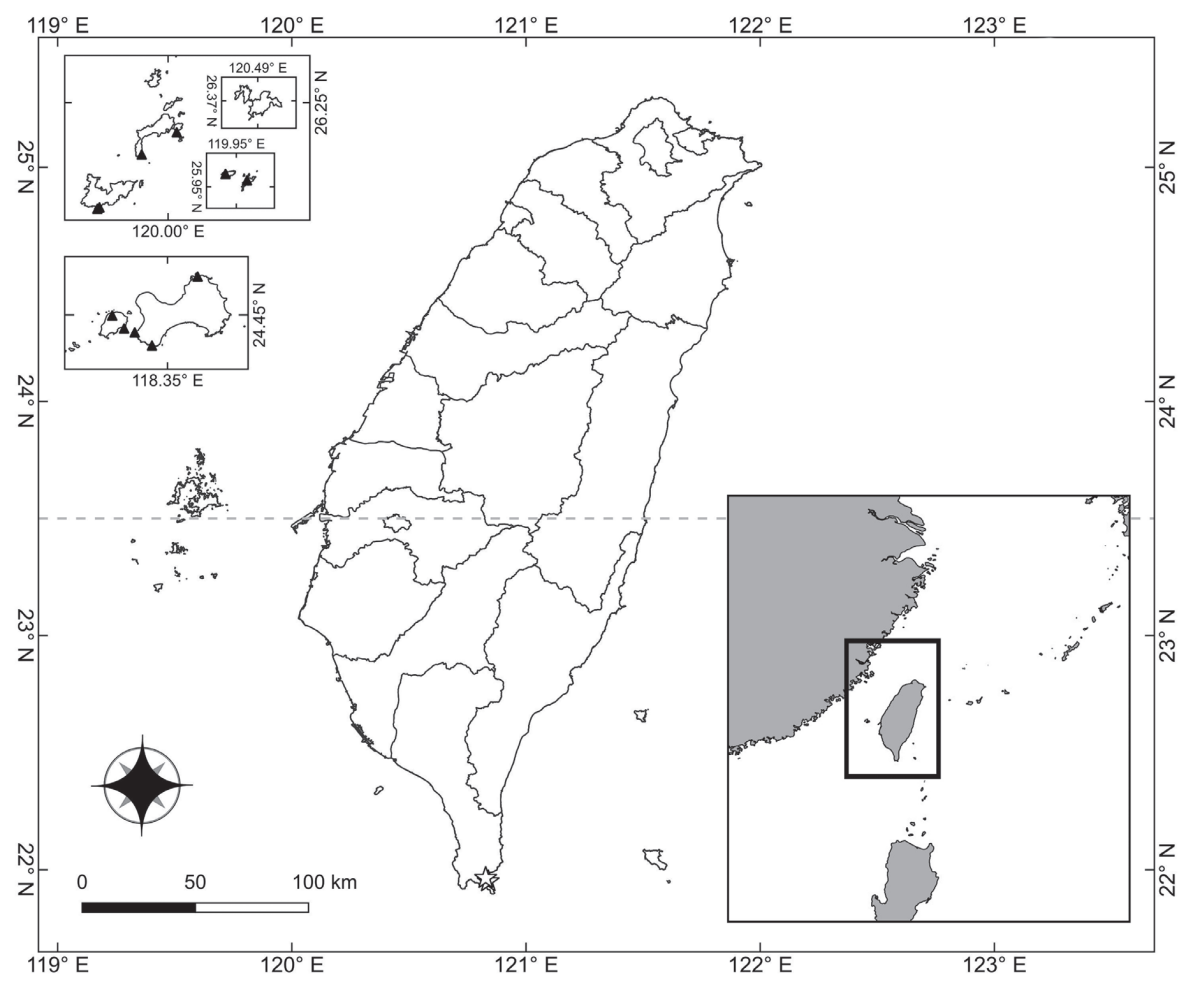
Distribution map of *Grewiakentingensis* Y.H.Tseng, Chih Y.Chang & C.Y.Lin (white star) and *G.piscatorum* Hance (black triangle) in Taiwan.

*Grewiakentingensis* is only found along the eastern coastline of Hengchun Peninsula. This species grows on elevated coral reefs and open coastal grasslands with intense sunlight. By contrast, *G.piscatorum* is found in coastal hills or islands in Fujian and Hainan provinces in China (Chun and Chang 1965; Chang 1987; Chang 1989; Tang et al. 2007). In Taiwan, *G.piscatorum* is only found in Kinmen and Lienchiang counties (Fig. 5).

### ﻿Key to *Grewia* in Taiwan modified from Liu and Lo (1993) and Chang et al. (2018)

**Table:** 

1a	Leaf bases cordate to rounded, usually oblique; inflorescences axillary; flowers bisexual only; sepals reflexed; fruits unlobed or 2-lobed	**2**
2a	Abaxial surface of leaves tomentose; petals and stamens turn from yellow to orange before withering; fruit surface tomentose	***G.eriocarpa***
2b	Abaxial surface of leaves nearly glabrous; petals and stamens turn from yellow to red before withering; fruit surface pubescent	***G.tiliifolia***
1b	Leaf bases cuneate, obtuse to rounded, seldom oblique; inflorescences terminal or rarely axillary; flowers unisexual or bisexual; sepals erect or slightly reflexed; fruits usually 2- to 4-lobed	**3**
3a	Procumbent shrubs; leaf blade length < 2 cm; gynodioecious; inflorescences terminal, 1–3(4–6) flowers per peduncle; stamens < 40 per flower	***G.kentingensis***
3b	Trees or erect to ascending shrubs; leaf blade length often > 2 cm; trioecious; inflorescences leaf-opposed, 4–15 flowers per peduncle, rarely axillary; stamens > 50 per flower	**4**
4a	Leaves densely covered with stellate hairs above and below	***G.rhombifolia***
4b	Leaves surfaces nearly glabrous above and below	**5**
5a	Small trees ca. 3–5 m tall; leaf blades ovate to elliptic, sometimes rhomboid-ovate, maximum length > 10 cm	**G.bilobavar.biloba**
5b	Erect to ascending shrubs ca. 1–2 m tall; leaf blades elliptic, obovate, or ovate to rhomboid-ovate, maximum length < 10 cm	***G.piscatorum***

### ﻿Taxonomic treatment

#### ﻿ Grewia kentingensis

Taxon classificationPlantaeMalvalesMalvaceae

Y.H.Tseng, Chih Y.Chang & C.Y.Lin sp. nov.

87979378-1FEA-58F4-AFC1-CF1A4D0954B9

urn:lsid:ipni.org:names:77357855-1

**Figure 6.**
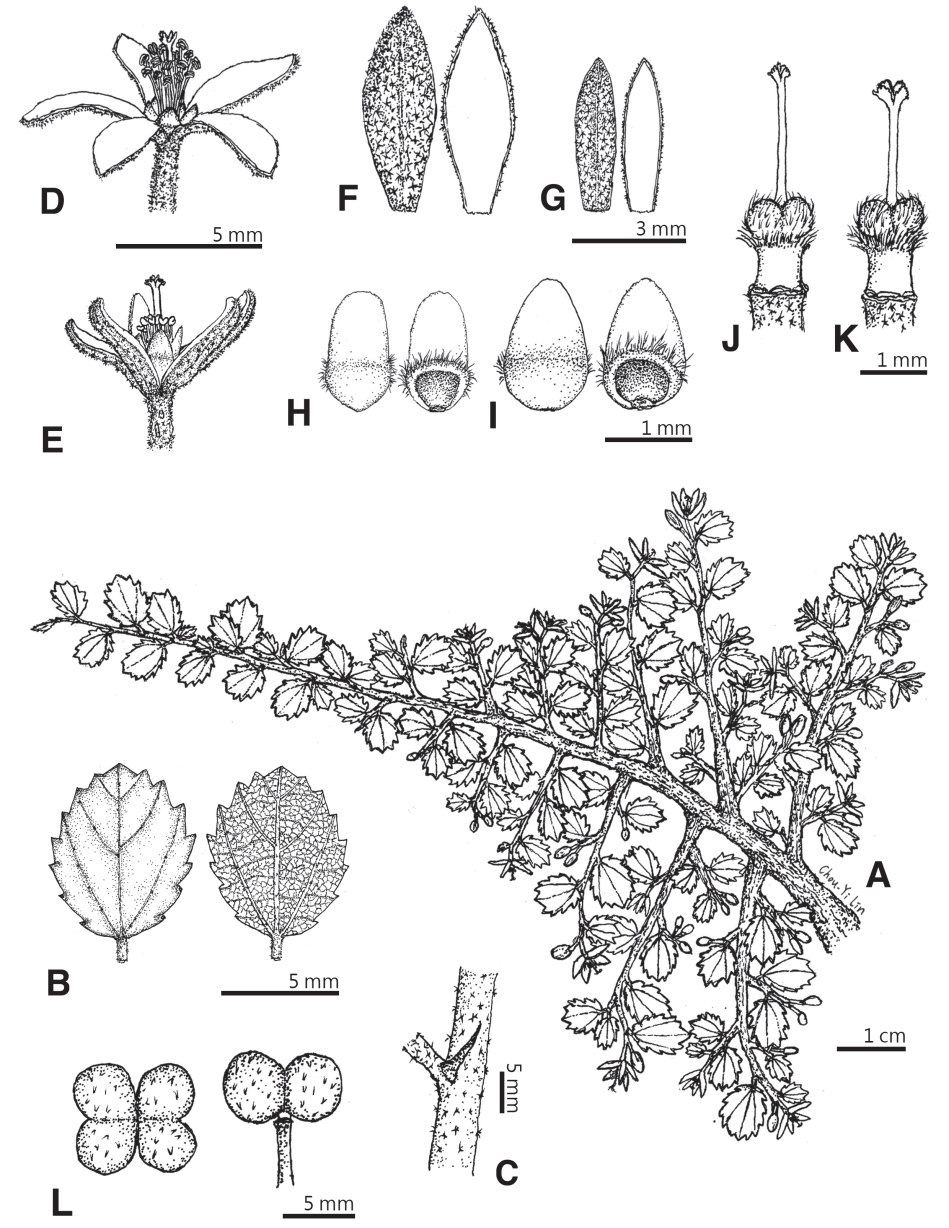
*Grewiakentingensis* Y.H.Tseng, Chih Y.Chang & C.Y.Lin. **A** habit **B** leaves **C** stipule **D** bisexual flower **E** female flower **F** sepals (bisexual) **G** sepals (female) **H** petals (bisexual) **I** petals (female) **J** gynoecium (bisexual) **K** gynoecium (female) **L** fruit (left: overhead view; right: lateral view).

**Figure 7.**
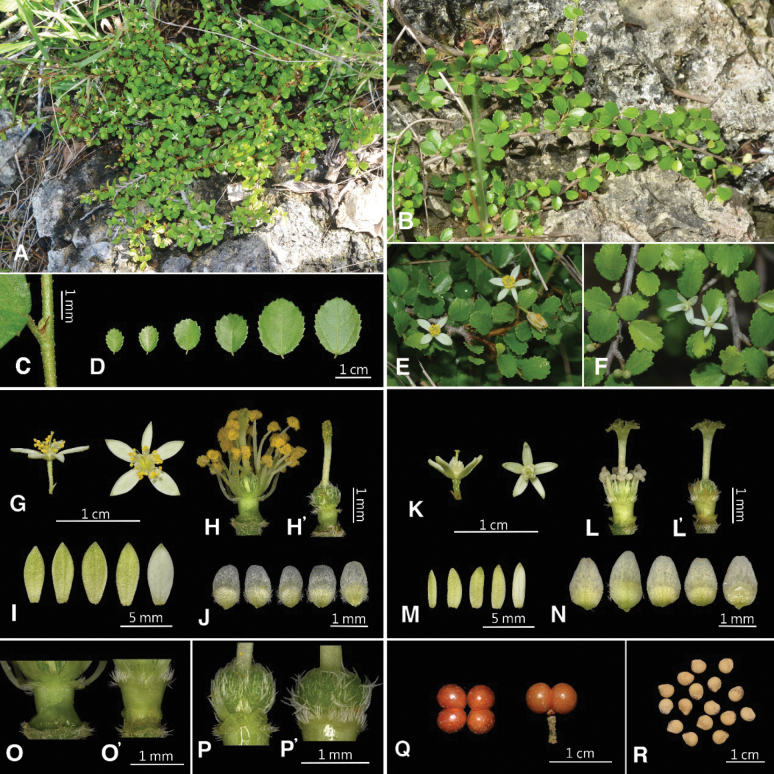
*Grewiakentingensis* Y.H.Tseng, Chih Y.Chang & C.Y.Lin. **A** habitat **B** habit **C** stipule **D** leaf blade variation **E** inflorescence (bisexual) **F** inflorescence (female) **G** bisexual flowers **H** bisexual flower (sepals and petals removed), **H**' gynoecium (bisexual) **I** sepals (bisexual) **J** petals (bisexual) **K** female flowers **L** female flower (sepals and petals removed), **L**' gynoecium (female) **M** sepals (female) **N** petals (female) **O** androgynophore (bisexual) **O**' androgynophore (female) **P** ovary (bisexual) **P**' ovary (female) **Q** fruit (left: overhead view; right: lateral view) **R** pyrenes.

**Figure 8.**
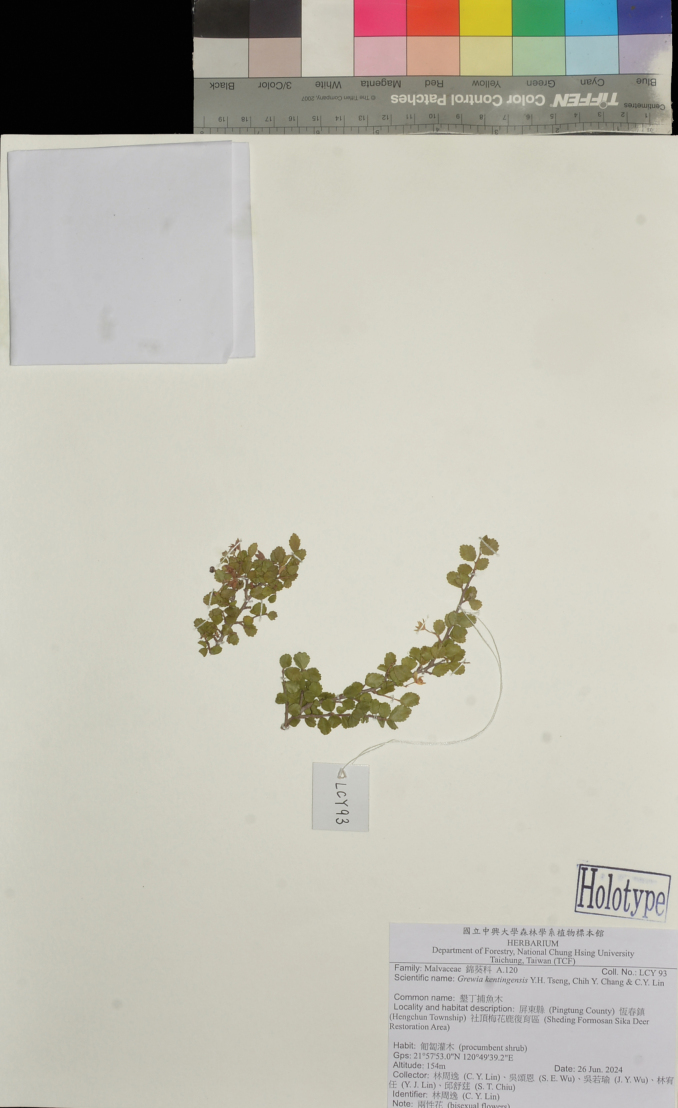
Holotype of *Grewiakentingensis* Y.H.Tseng, Chih Y.Chang & C.Y.Lin, *C. Y. Lin et al. 93* (TCF).

Figs 1A , 2A , 3A , 6 , 7 , 8

##### Diagnosis.

*Grewiakentingensis* is similar to *G.piscatorum* but can be distinguished by its procumbent (vs. erect to ascending) habit, smaller leaf size, serrate (vs. biserrulate) leaf margin, terminal (vs. often leaf-opposed) inflorescences, gynodioecious (vs. trioecious) breeding system, smaller flowers, fewer stamens, smaller fruits, and smaller pollen grains.

##### Type.

Taiwan. Pingtung County • Hengchun Township, Sheding Formosan Sika Deer Restoration Area, 154 m alt., 21°57'53.0"N, 120°49'39.2"E, 26 June 2024, *C. Y. Lin et al. 93* (holotype: TCF; isotype: TNM).

##### Description.

Procumbent shrubs to ca. 5 cm tall, stems rooting at the nodes; young branchlets puberulent, older branchlets nearly glabrous. Leaf blades broadly elliptic to elliptic, 0.4–1.6 cm long, 0.3–1.3 cm wide, apex rounded to obtuse, base cuneate to rounded, 3-nerved, margin serrate, nearly glabrous, stellate hairs sparsely distributed along the veins on both surfaces; petioles 0.4–1.3(–1.7) mm long, stellate hairs sparse; stipules linear, 0.4–1.2 mm long. Inflorescences terminal, cymose or flowers solitary; peduncles 0.7–2.7 mm long, 1 to 3(4 to 6) flowers, pedicels 0.9–4.9 mm long; bracts linear, 0.7–1.4(–1.8) mm long. Flowers bisexual or functionally unisexual (female), bisexual flowers 7.2–11.7 mm diam., female flowers 5.2–7.8 mm diam. Sepals (3)4 to 5, narrowly oblong to oblong, bisexual flowers 4.5–6.0 mm long, 1.4–2.3 mm wide; female flowers 3.2–4.8 mm long, 0.9–1.9 mm wide, apex acute, abaxial surface yellowish green, stellate–pubescent, adaxial surface white, glabrous. Petals (3)4 to 5, oblong to ovate, apex rounded, bisexual flowers 1.1–1.5 mm long, 0.6–0.9 mm wide, female flowers 1.2–1.6 mm long, 0.7–0.9 mm wide; nectaries present at the base of adaxial surface, 0.6–0.9 mm diam., surrounded by ciliate hairs. Androgynophore cylindrical, bisexual flowers 0.7–0.9 mm long; female flowers 0.8–0.9 mm long, glabrous, ciliate hairs only at the apex. Ovary globose to oblate, pubescent, bisexual flowers 0.8–1.0 mm diam.; female flowers 0.8–1.0 mm diam. Bisexual flowers with stamens 25 to 33, filaments white, glabrous, 1.2–3.2 mm long, anthers dehiscing longitudinally; style (1.8–)2.3–2.7 mm long, glabrous, stigma 4-lobed, each lobe dentate at apex. Female flowers with 15–20 stamens, filaments white, glabrous, 0.6–1.1 mm long, anthers white, always indehiscent; style 1.3–2.2 mm long, glabrous, stigma 4-lobed, each lobe dentate at apex. Fruits drupaceous, usually 4-lobed, fruit lobe 4.1–5.2 mm diam., globose, puberulent to nearly glabrous, red when mature.

##### Phenology.

Flowering from May to August and fruiting from June to September.

##### Distribution and habitat.

The species is endemic to Taiwan. *Grewiakentingensis* is sparsely distributed only on the elevated coral reefs and the coastal open grasslands at 50–200 m alt. along the eastern coastline of the Hengchun Peninsula. Commonly associated species include *Maytenusdiversifolia* (Maxim.) Ding Hou (Celastraceae), *Pandanusodorifer* (Forssk.) Kuntze. (Pandanaceae), PhoenixloureiroiKunthvar.loueiroi (Palmae), *Rostellulariahayatae* (Yamam.) S.S.Ying (Acanthaceae), *Galactiatashiroi* Maxim. (Fabaceae), and *Cirsiumalbescens* Kitam. (Compositae).

##### Chinese name.

kěn-dīng-bǔ-yú-mù (墾丁捕魚木)

##### Etymology.

The species epithet *kentingensis* refers to the type locality of Kenting in Hengchun Peninsula, Pingtung County, Taiwan.

##### Palynology.

Pollens grains are tricolporate, subprolate to prolate, 35.8–41.4 × 24.1–33.4 μm. Exine reticulate, with muri 0.6–0.8 μm wide and lumen 1.0–2.9 μm. Perforations present in the lumen, 0.04–0.24 μm.

##### Conservation status.

*Grewiakentingensis* has an extremely limited distribution on the Hengchun Peninsula and is known from 12 collections representing three populations. It has a geographic range in the form of an estimated EOO of 8 km^2^ (adjusted upward from 1.423 km^2^ following IUCN 2024) and an AOO of 8 km^2^. Even though the distribution of the species is located within the areas of Kenting National Park, its habitat is threatened by grazing, wildfires, and invasive plant species (*Cuscutacampestris* Yunck., *Stachytarphetajamaicensis* (L.) Vahl, and Bidenspilosavar.radiata (Sch. Bip.) J.A. Schmidt). Given these ongoing threats, we infer a continuing decline in the area, extent and quality of habitat. With respect to the most serious plausible threat of wildfire, the three occurrences represent one location, which falls within the limits for “Critically Endangered” status. *Grewiakentingensis* is therefore preliminarily assessed as “Critically Endangered” [CR B1ab(iii)+2ab(iii)] in accordance with the IUCN Red List Categories and Criteria (IUCN 2024). The above analysis is based on the specimens cited and georeferenced as shown in Table 4.

**Table 4.** Specimens and coordinates used for the analysis of the conservation status.

Latitude / Longitude	Specimens
21°56'52.3"N, 120°50'18.3"E	*T. C. Huang & S. F. Huang 13542*; *K. C. Chang 4893*, *4894*; *C. Y. Chang & C. H. Liu 397*; *C. Y. Lin et al. 69*
21°56'31.1"N, 120°49'37.9"E	*P. F. Lu 16921*; *T. C. Hsu 1743*; *C. M. Wang et al. 15470*; *C. F. Chen 4697*; *C. Y. Chang 30*
21°57'53.0"N, 120°49'39.2"E	*Y. H. Yu 7968*; *C. Y. Chang et al. 3236*; *C. Y. Lin et al. 93*

##### Specimens examined.

**Taiwan. Pingtung County** • Fongchueisha, 28 Jun 1988, *T. C. Huang & S. F. Huang 13542* (TAI) • Ibid., 23 Jul 2013, *K. C. Chang 4893, 4894* (CHIA) • Ibid., 29 Mar 2015, *C. Y. Chang & C. H. Liu 397* (TNM) • Ibid., 3 May 2024, *C. Y. Lin et al. 69* (TCF) • Longzaipu, 6 Sep 2008, *P. F. Lu 16921* (TAIF) • Ibid., 17 Sep 2008, *T. C. Hsu 1743* (TAIF) • Ibid., 23 Jul 2013, *C. M. Wang et al. 15470* (TNM) • Ibid., 14 Aug 2013, *C. F. Chen 4697* (TAIF) • Ibid., 12 Apr 2014, *C. Y. Chang 30* (PPI) • Sheding, 12 Aug 1995, *Y. H. Yu 7968* (TAIF) • Sheding Formosan Sika Deer Restoration Area, 15 Jan 2021, *C. Y. Chang et al. 3236* (TNM).

#### ﻿ Grewia piscatorum

Taxon classificationPlantaeMalvalesMalvaceae

Hance in Ann. Sci. Nat., Bot., sér. 5, 5. 208. 1866.

BE673D1D-687D-5801-B36D-31B82A816AB9

**Figure 9.**
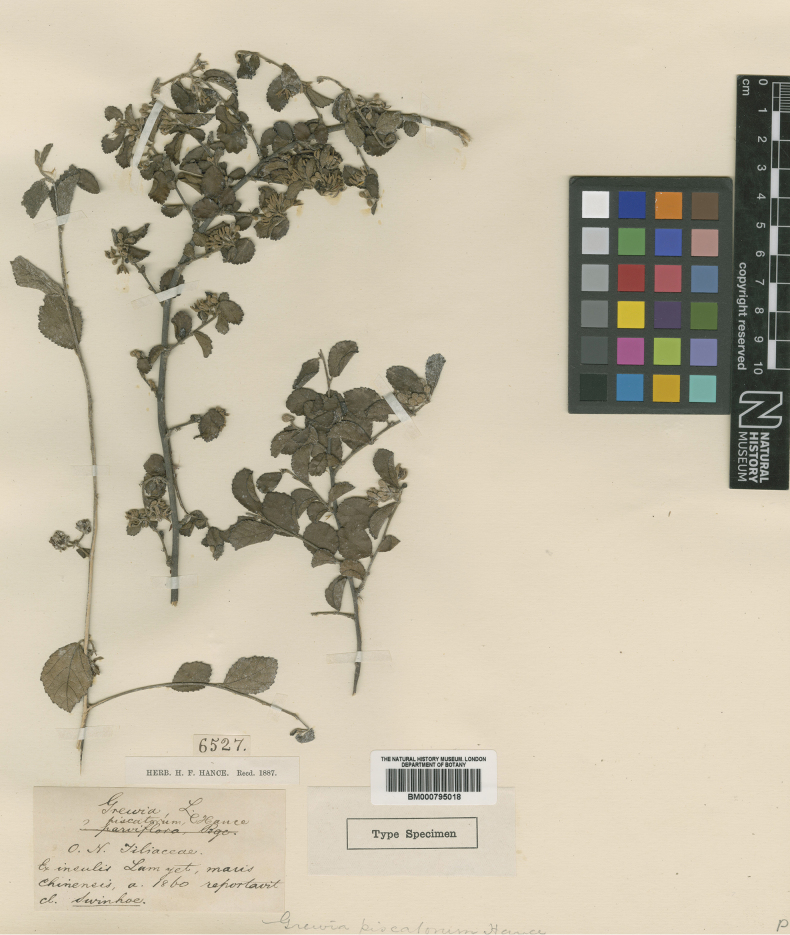
Lectotype of *Grewiapiscatorum* Hance, *Swinhoe s.n.*in Herb. Hance 6527 (BM000795018).

Figs 1B , 2B , 3B , 4 , 9

##### Type.

China • Fokien province: Lamyet island, 1860, *Swinhoe s.n.* in Herb. Hance 6527 (lectotype, designated here: BM [barcode BM000795018 as image!]; isolectotype, K [barcode K000686751 as image!]).

##### Notes.

In the protologue, Hance (1866) cited a Swinhoe collection that he assigned one of his own collection numbers (“Herb. propr., no. 6527”). Hance (1866) did not unequivocally state that he based the name on a single specimen and since there are duplicates of this Hance number in BM (BM000795018) (Fig. 9) and K (K000686751), we designate a lectotype for *Grewiapiscatorum*. The sheet selected is in Hance’s herbarium (BM), and it is the duplicate with relatively more leaves and flowers.

##### Description.

Erect to ascending shrubs, ca. 1–2 m tall; young branchlets scabrous, older branchlets nearly glabrous. Leaf blades elliptic, obovate, ovate to rhomboid-ovate, 0.7–7.8 cm long, 0.5–5.5 cm wide, apex acute, obtuse to rounded, base cuneate to obtuse, 3-nerved, margin serrulate to biserrulate, nearly glabrous, stellate hairs sparsely distributed along the veins on both surfaces; petioles 1.3–13.4 mm, sparsely stellate hairy; stipules linear, 2.4–5.1 mm long. Inflorescences leaf-opposed or rarely axillary, umbellate; peduncles 3.1–17.2 mm long, 6 to 15 flowers, pedicels 3.5–8.8 mm long; bracts linear, 1.4–2.6(–3.0) mm long. Flowers bisexual, functionally unisexual male or functionally unisexual female; bisexual flowers 14.8–18.5(–20.2) mm diam.; male flowers 14.2–18.8 mm diam.; female flowers 9.4–14.6 mm diam. Sepals (4)5(6), narrowly oblong to oblong, bisexual flowers 7.5–10.8 mm long, 2.4–4.1 mm wide, male flowers 7.3–8.4 mm long, 2.1–3.0 mm wide, female flowers 4.5–5.7 mm long, 1.4–2.0 mm wide, apex acute, abaxial surface yellowish green, stellate–pubescent, adaxial surface white, glabrous. Petals (4)5(6), oblong to ovate, apex rounded, bisexual flowers 2.2–3.3 mm long, 0.9–1.2 mm wide, male flowers 2.1–2.9 mm long, 0.9–1.2 mm wide, female flowers 1.5–2.5 mm long, 0.6–1.0 mm wide; nectaries present at the base of adaxial surface, 0.8–1.3 mm diam., surrounded by ciliate hairs. Androgynophore cylindrical, bisexual flowers 1.1–1.6 mm long; male flowers 1.1–1.5 mm long; female flowers 0.9–1.2 mm long, glabrous, ciliate hairs only at the apex. Ovary globose to oblate, pubescent, bisexual flowers 1.2–1.4 mm diam.; male flowers 1.2–1.5 mm diam.; female flowers 0.9–1.3 mm diam. Bisexual flowers with 103–118 stamens, filaments white, glabrous, 2.4–6.8 mm long, anthers dehiscing longitudinally; style 3.6–5.4 mm long, glabrous, stigma 4-lobed, each lobe dentate at apex. Male flowers with 70–88 stamens, filaments white, glabrous, 2.1–4.7 mm long, anthers dehiscing longitudinally; style 3.1–4.1 mm long, glabrous, stigma 4-lobed, each lobe dentate at apex. Female flowers with 58–102 stamens, filaments white, glabrous, 0.5–1.5 mm long, anthers white, always indehiscent; style 1.8–2.6 mm long, glabrous, stigma 4-lobed, each lobe dentate at apex. Fruits drupaceous, usually 4-lobed, fruit lobe 4.4–6.4 mm diam., globose, puberulent to nearly glabrous, red when mature.

##### Phenology.

Flowering from May to September and fruiting from June to October.

##### Distribution and habitat.

Distributed in Fujian Province and Hainan Province in China and in Taiwan on the coastal areas of Kinmen and Lienchiang Counties.

##### Palynology.

Pollens grains are tricolporate, prolate, 42.8–47.8 × 27.4–31.0 μm. The exine is reticulate, with muri 0.4–0.7 μm wide and lumen size 1.1–4.0 μm. Perforations present in the lumen, 0.04–0.26 μm.

##### Specimens examined.

**China. Fujian Province** • Fuzhou City, Tangyu, 24 May 1964, *T. H Chen 493* (AU) • Fuzhou City, Yemayu, 31 May 1964, *T. H Chen 515* (AU); Fuzhou City, Zhuyu, 17 May 1963, *T. H Chen 1561* (AU) • Xiamen City, Nanputuo, 20 Jul 1954, *J. C. Lin 3857* (AU). **Hainan Province** • Dongfang City, seacoast of Dingjiao village, 20 Aug 1936, *S. K. Lau 27727*, *27730* (IBSC); Sanya City, Xigu island, 3 Aug 2018, *H. L. Hou 92038* (AU). **Taiwan. Kinmen County** • Brave Fortress, 24 Jun 2017, *S. W. Chung 12988* (TAIF) • Caicuo Trail, 2 Jun 2019, *C. T. Lu et al.* 2515 (TNM) • Doumen to Taiwu Mountain, 22 Jul 2007, *C. M. Wang 10497* (TNM) • Fengshang, 20 May 2009, *J. S. Shiu & Y. L. Hsueh H315* (CHIA) • General’s Spring, 28 May 2023, *C. Y. Lin 22* (TCF) • Jiugong Pier, 25 May 2002, *C. M. Wang 5611* (TNM) • Ibid., 7 Sep 2021, *C. Y. Chang et al. 3539* (TNM)Ibid., 28 May 2023, *C. Y. Lin 17* (TCF) • Jiugong Tunnel to shore-line road, 28 May 2023, *C. Y. Lin 20* (TCF) • Liaoluo, 22 May 2017, *T. C. Hsu 9131* (TAIF) • L36 Fortress, 25 May 2024, *C. Y. Lin et al. 85* (TCF) • Mashan Observation Post, 26 Aug 2015, *C. Y. Chang et al.* 724 (TCF, TNM) • Ibid., 29 May 2023, *C. Y. Lin 24* (TCF) • Paichushan to Huotou, 10 Jun 1999, *S. T. Chiu 5386* (TNM) • Qilinshan, 25 May 2024, *C. Y. Lin et al. 86* (TCF) • Qingyuan Lake, 9 Aug 2002, *I. H. Chiang 120* (TAI) • Shuitou Pier, 28 May 2023, *C. Y. Lin 21* (TCF) • Taiwu Mountain, 27 Jul 2005, *K. C. Chen s.n.* (TAIF) • Ibid., 15 Jun 2006, *C. H. Chen 7283* (TNM) • Ibid., 4 Jun 2016, *S. W. Chung 12534* (TAIF) • Taiwu nursery, 22 Jul 2007, *C. M. Wang 10519* (TNM) • Tashan, 14–17 May 2010, *F. Y. Lu et al. H2794* (CHIA) • Tzuhu, 25 Jul 2005, *K. C. Chen s.n.* (TAIF) • Tzuti, 25 Jul 2005, *K. C. Chen s.n.* (TAIF) • Wuhu Mountain, 13–16 Jul 2009, *F. Y. Lu et al. 1015* (CHIA) • Xiyuan beach to Chengkung Fortress, 29 May 2023, *C. Y. Lin 23* (TCF) • Zhaishan Tunnel, 25 May 2024, *C. Y. Lin 80* (TCF). **Lienchiang County** • Beihai Tunnel, 19 Jul 2008, *C. M. Wang 12180* (TNM) • Chengkungshan, 27 Aug 2007, *T. W. Hsu 13020* (TAIE) • Chiukueishan, 27 Aug 2001, *Y. L. Huang et al. 816* (TAI) • Daping to lighthouse, 30 May 1994, *T. Y. Liu & H. L. Ho 653* (TNM) • Dapu Inscription, 9 Sep 2007, *C. M. Wang & C. P. Lu 10690* (TNM) • Fuzheng, 30 Jul 1999, *S. H. Su 624* (TAI) • Jinsha, 1 Jul 1999, *S. H. Su 352* (TAI) • Dawo Mountain, 16 Sept 2014, *S. W. Chung 11782* (TAIF) • Kunqiu Trail, 17 Oct 2017, *C. Y. Chang 1473* (TCF, TNM) • Leishan, 29 Aug 2007, *T. W. Hsu 13079* (TAIE).

## Supplementary Material

XML Treatment for Grewia kentingensis

XML Treatment for Grewia piscatorum

## ﻿Additional information

### Author ORCIDs

Chou-Yi Lin https://orcid.org/0009-0009-2076-9881

Chih-Yi Chang https://orcid.org/0000-0002-4226-9847

Hsy-Yu Tzeng https://orcid.org/0000-0003-0952-6255

Yen-Hsueh Tseng https://orcid.org/0000-0003-3644-1863

### Data availability

All of the data that support the findings of this study are available in the main text.
